# Robot‐Assisted Upper‐Limb Rehabilitation After Stroke: A Systematic Review and Meta‐Analysis of Cortical Reorganization and Neuroplasticity Biomarkers

**DOI:** 10.1155/np/9282578

**Published:** 2026-06-05

**Authors:** Rocco Salvatore Calabrò, Andrea Calderone, Lilla Bonanno, Angelo Quartarone, Antonino Naro, Alessandro Marco De Nunzio, Serena Filoni

**Affiliations:** ^1^ Neurorehabilitation Unit, IRCCS Bonino-Pulejo Center for Neurolesions, Messina, Italy; ^2^ Stroke Unit, University Hospital G. Martino, Messina, Italy; ^3^ Department of Health, LUNEX University of Applied Sciences, Differdange, Luxembourg; ^4^ Research Department, Luxembourg Health and Sport Sciences Research Institute, Differdange, Luxembourg; ^5^ Neurorehabilitation Unit, IRCCS House for the Relief of Suffering, San Giovanni Rotondo, Italy

**Keywords:** corticospinal excitability, motor-evoked potentials, robot-assisted rehabilitation, stroke, transcranial magnetic stimulation, upper extremity

## Abstract

**Background:**

Robot‐assisted upper‐limb rehabilitation is widely used after stroke, but links to neuroplasticity biomarkers remain uncertain.

**Objective:**

To synthesize biomarker evidence after robot‐assisted upper‐limb training and quantify post‐intervention effects versus non‐robot comparators.

**Methods:**

We searched PubMed, Web of Science, Embase, Cochrane Library, EBSCOhost, and Scopus from inception to December 31, 2025. Randomized controlled trials (RCTs) and nonrandomized intervention studies enrolling adults with stroke were eligible if they reported at least one neuroplasticity biomarker. Prespecified comparison classes were used; only the primary contrast (robot‐assisted training vs. non‐robot control) was pooled at post using DerSimonian–Laird random‐effects models. Effects were expressed as standardized mean differences (SMDs; Hedges’ g) or mean differences (MDs) with 95% confidence intervals (CIs).

**Results:**

Fifty‐five studies, including 24 RCTs, were included. Four RCTs contributed to the primary quantitative synthesis, and each pooled endpoint included two or three trials. Robot‐assisted training was associated with improved ipsilesional resting motor threshold (RMT; SMD 1.77, 95% CI: 0.46–3.08; *k* = 2) and upper‐limb impairment (Fugl–Meyer assessment‐upper extremity [FMA‐UE]; MD 4.48 points, 95% CI: 0.33–8.62; *k* = 3). Effects were uncertain for ipsilesional motor‐evoked potential (MEP) amplitude (SMD 0.52, 95% CI: −0.31–1.34; *k* = 2) and activities of daily living (ADL). Heterogeneity was moderate to substantial, prediction intervals crossed the null, and certainty was low or very low.

**Conclusions:**

Robot‐assisted training may improve impairment and corticospinal excitability, but randomized biomarker evidence remains sparse and heterogeneous. Biomarkers should be interpreted as exploratory observations, not validated surrogates or causal evidence of cortical restitution.

## 1. Introduction

Stroke remains a leading contributor to long‐term disability, with the Global Burden of Disease Study 2019 estimating 12.2 million incident strokes and 101 million stroke survivors worldwide in 2019 [1]. Upper‐limb paresis limits independence and participation, and many individuals do not regain dexterous hand use despite access to rehabilitation [2]. Training dose, task specificity, and practice quality shape recovery potential, yet conventional services often cannot deliver sufficient repetitions with appropriate progression and feedback [2].

Robot‐assisted upper‐limb rehabilitation aims to address these constraints. Devices can deliver high‐volume, task‐oriented practice with standardized progression while capturing kinematic and kinetic performance metrics. Clinical syntheses have evaluated robot‐assisted therapy mainly through functional outcomes and activities of daily living (ADL), with conclusions influenced by device diversity, treatment dose, comparator intensity, and patient severity [3–5]. Mechanistic outcomes have been reported inconsistently and are rarely pooled quantitatively, which limits inference about how robot‐mediated practice relates to neural repair or reorganization.

Motor improvement after stroke can reflect compensation learning, recovery learning, or both [6]. Compensation learning prioritizes task success through alternative strategies, such as trunk recruitment or proximal substitution. Recovery learning aims to restore impaired motor patterns and regain degrees of freedom within the redundant arm, which supports adaptability in daily contexts [6]. Arm function relies on coordinated control across multiple joints and effectors, which enables flexible solutions for the same goal [7]. Task designs that permit success through restricted solutions may reinforce compensation, while designs that shape exploration and stabilize key task variables may support the reacquisition of missing degrees of freedom [6, 7].

Motor learning theory links the practice structure to neural change. Error‐based learning updates internal models when sensory prediction errors are available and appropriately scaled [7]. Use‐dependent learning reflects repetition‐driven plasticity that stabilizes preferred actions over time [8]. Reinforcement learning shapes behavior through outcome‐based signals when error information is limited or when the task space is redundant [9]. These mechanisms can interact during rehabilitation, and device control policies can shift the dominant learning signal by manipulating errors, assistance, task success, and exploration [7–9].

Robotic systems vary in form and control, which may influence learning demands and neuroplastic response. End‐effector robots apply forces at a distal contact point and constrain interaction to the endpoint [10]. Exoskeletons align with limb segments and can target joint‐level assistance or resistance to address multijoint coordination [10]. Wearable hand devices and soft robotic gloves support grasp and release in more ecological contexts [10]. Control strategies include assist‐as‐needed policies that adjust support based on performance [11], impedance and admittance control that tune haptic guidance or resistance [11, 12], and approaches that amplify errors or impose force fields to drive adaptation and exploration [11, 13]. Reinforcement‐based task structures can reward recovery‐relevant solutions that are not directly constrained by task goals [9].

Neuroplasticity biomarkers complement behavioral endpoints by indexing corticospinal and network physiology. Transcranial magnetic stimulation (TMS) can probe corticospinal excitability and intracortical circuits relevant to pathway integrity and training responsiveness [14]. Electroencephalography (EEG) can quantify oscillatory dynamics, laterality, and network interactions during movement or motor imagery [15]. Structural and functional neuroimaging can characterize reorganization within distributed motor systems, and diffusion‐based corticospinal measures relate to impairment severity [16]. Hybrid protocols that combine robotics with neurofeedback or brain–computer interfaces may individualize training, yet attribution becomes challenging when multiple technologies co‐deliver therapy.

This systematic review and meta‐analysis focuses on stroke because mechanistic evidence linking upper‐limb robotics to neurophysiological or neuroimaging outcomes is concentrated in stroke populations, while evidence in other neurological conditions is sparse. Existing reviews mainly synthesize clinical outcomes and do not provide a dedicated quantitative synthesis of neuroplasticity biomarkers with a prespecified plan to handle biomarker families, device classes, stroke stage, and hybrid interventions. The present review aims to address this gap by identifying robot‐assisted upper‐limb intervention studies in adults with stroke that include at least one neurophysiological or neuroimaging outcome consistent with neuroplastic change and then pooling compatible randomized evidence when feasible. The planned synthesis also evaluates how device class and control strategy map onto motor learning mechanisms that plausibly support cortical reorganization, while separating standalone robotics from hybrid approaches through subgroup and sensitivity analyses.

## 2. Materials and Methods

### 2.1. Protocol Registration and Reporting

The protocol was registered in PROSPERO on January 18, 2025 (CRD42025635558) and updated on January 10, 2026 (Version 2.0). An additional administrative update dated February 1, 2026 was submitted to align the public record with the final manuscript after iterative drafting and internal revisions; this update clarified wording and transparency items without changing the core eligibility criteria, prespecified key outcomes for quantitative synthesis, or the planned primary analyses. Therefore, the record should not be interpreted as fully prospective in the strictest sense, but the version history provides a date‐stamped audit trail of amendments. Reporting followed PRISMA 2020 guidance and checklist [17] (Supporting Information 1: Material S1). Full reproducibility details, decision rules, and full search strategies are provided in Supporting Information 2: Material S2 (Appendix S1).

### 2.2. PICO Framework

The review question was structured using the PICO framework by defining the target population, intervention categories, comparators, and prespecified outcomes. The population included adults aged 18 years or older with ischemic or hemorrhagic stroke and upper‐limb motor impairment. Interventions were robot‐assisted upper‐limb rehabilitation delivered through end‐effector robots, exoskeletons, wearable hand devices, or soft robotic gloves. For quantitative synthesis, studies were grouped into prespecified contrasts: (i) robot‐assisted rehabilitation versus non‐robotic rehabilitation (dose‐matched conventional therapy or usual care) (primary); (ii) robot‐assisted rehabilitation plus an adjunct versus the same robotic training alone (secondary); and (iii) brain computer interface (BCI)‐guided robot training versus sham‐BCI or robot‐only training (exploratory). Eligible outcomes required at least one neurophysiological or neuroimaging measure consistent with neuroplastic change, including TMS metrics, EEG features, and neuroimaging outcomes from functional magnetic resonance imaging (fMRI), functional near‐infrared spectroscopy (fNIRS), or diffusion‐based structural imaging. Single‐group pre‐post intervention studies without a comparator were eligible for mapping when a defined robot‐assisted upper‐limb intervention was delivered, and pre‐post neuroplasticity biomarkers had a clear temporal link to the intervention period.

### 2.3. Inclusion Criteria

Eligible studies enrolled adults with stroke and reported a robot‐assisted upper‐limb intervention delivered as a therapeutic component intended to improve motor performance. Study designs included randomized controlled trials (RCTs) and nonrandomized intervention studies. Nonrandomized controlled intervention studies and single‐group pre‐post intervention studies (including feasibility/pilot studies) were eligible when biomarker data were extractable. Studies enrolling mixed neurological diagnoses were eligible only if stroke‐only data were reported separately or could be obtained from the authors. Observational designs were eligible only when a defined robotic intervention was delivered and pre‐post neuroplasticity outcomes were reported with a clear linkage to the intervention period. Studies were required to report at least one neurophysiological or neuroimaging outcome that operationalized neuroplasticity as measurable change in neural activity, network organization, corticospinal function, or structural connectivity. For the primary meta‐analysis, randomized trials were eligible only when they compared robot‐assisted training with a non‐robot comparator; trials where both arms received robotics were retained but analyzed separately by contrast class. Full‐text articles published in English were eligible. Hybrid interventions that combined robotics with virtual reality (VR), BCI control, or neurofeedback were eligible when robot‐assisted training remained the primary therapeutic driver, defined by the robot delivering the core movement practice and dosage. Sensitivity analyses were prespecified to evaluate robustness after excluding hybrid interventions.

### 2.4. Exclusion Criteria

Studies were excluded when participants did not have stroke, when the robotic device targeted the lower limb only, or when the device served solely as an assessment tool without a therapeutic training program. Studies were excluded when neuroplasticity outcomes were absent or limited to peripheral physiology without an explicit neural interpretation. Articles were excluded when they did not provide original clinical data suitable for synthesis, including narrative reviews, systematic reviews, meta‐analyses, guidelines, editorials, letters, commentaries, study protocols, and conference abstracts without full methodological reporting and extractable results. Case reports and cross‐sectional studies without an intervention were excluded. Non‐English full‐text articles were excluded to ensure consistent interpretation of neurophysiological and neuroimaging methods and to allow complete, reproducible data extraction; this restriction also reduced the risk of misclassifying biomarker definitions and analytic choices that can be highly sensitive to nuanced reporting.

### 2.5. Reporting Standard and Search Strategy

PubMed, Web of Science, Embase, Cochrane Library, EBSCOhost, and Scopus were searched from inception through December 31, 2025, and final searches were executed on January 12, 2026. Controlled vocabulary (MeSH/Emtree) was adapted per database and supplemented with free‐text keywords without time restrictions. Three core strings captured stroke, upper‐limb robotics, and modality terms for (1) TMS/EEG, (2) fMRI/diffusion imaging, and (3) fNIRS.

### 2.6. Study Selection

Two reviewers (Rocco Salvatore Calabrò and Andrea Calderone) independently screened titles/abstracts and then assessed full texts using predefined eligibility criteria. Decisions were made independently at each stage, and discrepancies were resolved by discussion to reach consensus; when consensus was not achieved, a third reviewer (Serena Filoni) adjudicated. At the full‐text stage, disagreements were resolved by reapplying the prespecified criteria to each report. Agreement statistics were computed for each screening stage to quantify consistency. Interrater agreement was substantial, with Cohen kappa values of 0.71 for title/abstract screening and 0.75 for full‐text eligibility assessment. The PRISMA flow diagram is presented in the results.

### 2.7. Data Extraction and Outcomes

Two reviewers (Rocco Salvatore Calabrò and Andrea Calderone) independently extracted design, participant, and stroke characteristics; intervention/comparator details; and outcomes, with cross‐checking for accuracy. Authors were contacted for missing or unclear data. Primary outcomes were neuroplasticity biomarkers, with key outcomes selected a priori for quantitative synthesis: ipsilesional resting motor threshold (RMT) (primary) and ipsilesional motor‐evoked potential (MEP) amplitude (key secondary) at immediate post‐intervention. Secondary clinical outcomes were the Fugl–Meyer assessment‐upper extremity (FMA‐UE) total and the Barthel index/modified Barthel index at post, when available. For TMS, RMT was prioritized over active motor threshold when both were reported, and unconditioned single‐pulse MEP amplitude was extracted where possible; when multiple muscles/conditions were reported, we extracted the trial‐defined primary measure. Post was defined as the first assessment closest to the end of intervention; follow‐up was grouped as FU1 (1–3 months) and FU2 (≥6 months). Change scores were preferred; post values were used otherwise. Additional validated upper‐limb/activity measures (e.g., action research arm test and wolf motor function test), ADL scales, kinematic performance metrics, adherence, and adverse events were extracted when reported to support clinical interpretation and safety reporting.

### 2.8. Risk of Bias, Data Synthesis, and Statistical Analysis

Randomized trials were evaluated using RoB 2 across five domains [18], and nonrandomized intervention studies using ROBINS‐I [19], by two independent reviewers (Rocco Salvatore Calabrò and Andrea Calderone) with consensus resolution.

Meta‐analysis was planned when ≥2 randomized trials reported compatible outcomes from comparable tasks, timepoints, and metrics. The primary quantitative synthesis pooled robot‐assisted rehabilitation versus non‐robot comparators; adjunct‐versus‐robot‐only and BCI‐versus‐sham/robot‐only contrasts were synthesized separately and were not combined with the primary contrast. Random‐effects models were the default for pooling using the DerSimonian–Laird estimator with inverse‐variance weighting [20], with fixed‐effect models examined in sensitivity analyses. Mean differences (MDs) were used for directly comparable units, whereas Hedges’ *g* was used when scales/units or acquisition conditions differed materially [21]. When change‐score variances required pre–post correlations, we imputed *r* = 0.50 as the primary value and explored *r* = 0.25 and *r* = 0.75 in sensitivity analyses. Effect direction was aligned so that positive values indicated improvement; outcomes where lower values indicated improvement were sign‐reversed before pooling. Missing SDs were derived from SEs, confidence intervals (CIs), *p*‐values, or test statistics when possible; otherwise, imputation from similar studies/timepoints was documented. Heterogeneity was summarized using *I* ^2^ and *τ* ^2^, and prediction intervals were reported when feasible [22]. Prespecified subgroup factors included robot class, stroke stage, training dose, biomarker family, and hybrid versus standalone interventions. Sensitivity analyses compared fixed‐ and random‐effects models, excluded high‐risk‐of‐bias studies and hybrids, and performed leave‐one‐out analyses. Multiarm trials with shared controls were handled by combining similar robot arms or splitting the control group across comparisons. Narrative synthesis followed SWiM guidance [23]; studies were grouped by biomarker family and design, and effects were summarized quantitatively when appropriate or by direction of effect when not. Nonrandomized intervention studies were synthesized cautiously due to confounding.

To preserve interpretability across study designs, randomized and nonrandomized evidence were not combined in any pooled estimate. RCTs were used to estimate comparative intervention effects when outcomes, timepoints, and contrasts were sufficiently compatible. Non‐RCTs and single‐group pre‐post studies were retained to map biomarker modalities, describe mechanistic signals, and identify hypothesis‐generating biomarker–behavior associations, but these designs were not used to strengthen causal claims because confounding, spontaneous recovery, concurrent rehabilitation, and selective outcome reporting could plausibly account for observed changes. Accordingly, nonrandomized findings were summarized narratively and interpreted as exploratory evidence.

### 2.9. Certainty of Evidence and Publication Bias

The certainty of evidence was assessed per outcome using the GRADE domains of risk of bias, inconsistency, indirectness, imprecision, and publication bias [24]. Randomized evidence started at high certainty and was downgraded by one level for serious concerns or by two levels for very serious concerns within any domain. Risk of bias downgrading was guided by RoB 2 judgments and by whether limitations were likely to materially influence effect estimates. Inconsistency considered heterogeneity magnitude, overlap of CIs, and plausibility of a common direction of effect. Indirectness reflected departures from the review question in the population, intervention implementation, comparator intensity, biomarker acquisition, or outcome definition. Imprecision considered CI width and whether intervals included both trivial and meaningful mechanistic or clinical effects, alongside available information size when feasible. A summary of findings table was prepared for RMT, MEP amplitude, FMA‐UE, and Barthel/modified Barthel at post. Funnel plots and Egger regression were planned when ≥10 studies contributed [25]. Evidence profiles and downgrade rationales are provided in Supporting Information 2: Material S2 (Appendix S1).

## 3. Results

**Figure 1:**
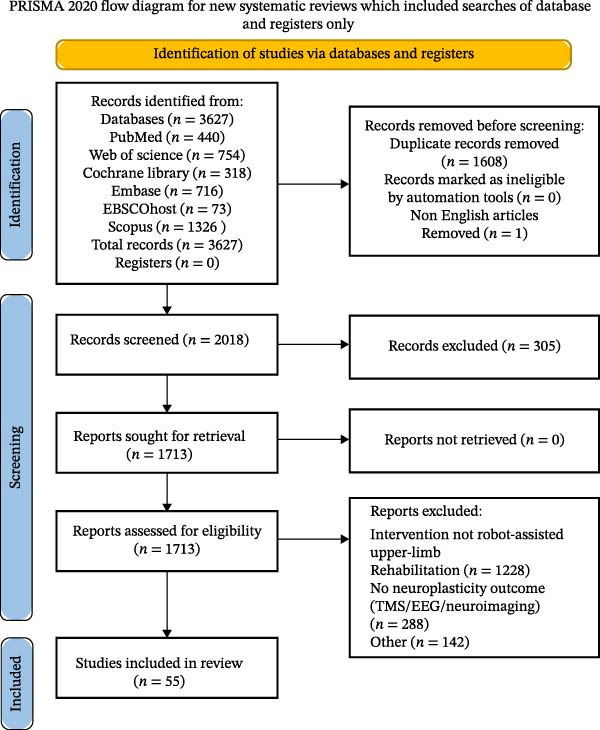
PRISMA 2020 flow diagram.

Database searches yielded 3627 records across six sources, with no records identified from registers. Duplicate removal excluded 1608 records, and one non‐English record was removed before screening. Title/abstract screening proceeded on 2018 records and excluded 305 records for ineligible study design. Full texts were sought for 1713 reports, and all were retrieved; 1658 were excluded at the eligibility assessment. Fifty‐five studies were included in the final review overall [26–80]. The study selection process is shown in Figure 1 [17].

### 3.1. Included Evidence and Clinical Context

The included evidence comprised 24 RCTs and 31 nonrandomized intervention studies evaluating upper‐limb robot‐assisted rehabilitation after stroke with at least one neuroplasticity biomarker outcome [26–80]. Study objectives ranged from estimating clinical effects to characterizing training‐related changes in corticospinal excitability, oscillatory dynamics, or network‐level measures. Participants were adults with ischemic or hemorrhagic stroke and upper‐limb motor impairment, with cohorts spanning subacute and chronic stages, as defined within individual reports. Baseline severity, lesion descriptions, and concomitant impairments were variably reported, limiting consistent stratification by biological capacity for recovery. Post‐intervention assessment was the dominant endpoint for both clinical and biomarker outcomes, whereas follow‐up was less frequent and commonly limited to selected measures rather than a harmonized panel [70, 72]. Supporting Information 3: Material S3 provides the full study‐level extraction summary.

Robotic interventions were heterogeneous in device architecture and training targets. End‐effector platforms and hand‐focused systems were common, and exoskeleton‐based approaches, wearable hand devices, soft robotic gloves, and neural‐guided robotic paradigms were also represented [29, 66, 71]. Intervention content ranged from proximal reaching to distal hand opening/closing or combined programs integrating multiple effectors. Assist‐as‐needed and adaptive difficulty paradigms were frequently used, often embedded in feedback‐rich or game‐like training environments. Comparator conditions varied substantially, spanning dose‐matched conventional therapy, structured non‐robot training, and usual care rehabilitation. Background therapy and co‐interventions were sometimes described in general terms, and quantitative documentation of comparator intensity was inconsistently reported, which is relevant when interpreting incremental effects attributable to robotics versus the broader rehabilitation program.

A subset of randomized evidence was eligible for the prespecified quantitative synthesis focused on robot‐assisted training versus non‐robot control. Eight RCTs contributed data to at least one prespecified post‐intervention key outcome across the three prespecified comparison classes [26, 27, 29, 44, 47, 55, 71, 74]. Four RCTs met criteria for the primary pooled contrast (robot‐assisted training versus non‐robot control at immediate post‐intervention), contributing to at least one key pooled outcome [26, 27, 44, 71]. Key characteristics of these trials, stroke stage, device class, comparator type, dose, samples analyzed at post, and outcomes contributed, are summarized in Table 1 [26, 27, 44, 71]. Trials in which both arms received robotics (robot+adjunct versus robot‐only) or in which robotic practice was identical and only a guidance/contingency component differed were retained for structured description but were not combined with the primary contrast because such comparisons estimate incremental effects on top of robotics rather than the effect of robotics versus non‐robot care.

**Table 1:** Characteristics of randomized trials contributing to the primary robot vs. non‐robot quantitative synthesis (post‐intervention).

Study (author, year)	Stroke stage	Robot class/device	Comparator	Dose	*N* robot/*N* control (post; analyzed)	Key outcomes contributed	Notes
Calabrò et al. 2019 [26]	Chronic (≥6 months post‐stroke)	End‐effector: Amadeo (tyromotion GmbH)	Dose‐matched: conventional hand therapy (OT) matched to robot training dose	40 × 45 min (1800 min; 30 h)	25/25	MEP; FMA‐UE	Multiarm: N; Hybrid: N
Singh et al. 2021 [27]	Subacute/early chronic (3–24 months post‐stroke)	Exoskeleton (hand): Electromechanical robotic hand exoskeleton (in‐house prototype)	Dose‐matched: conventional upper‐limb physiotherapy matched to robot training dose	20 × 45 min (900 min; 15 h)	12/11	RMT; MEP; FMA‐UE; Barthel/MBI	Multiarm: N; Hybrid: N
Tang et al. 2023 [44]	Subacute (2 weeks–3 months)	End‐effector: Burt (ESTUN Inc., Nanjing, China)— bilateral end‐driven system (two devices + cable)	Dose‐matched: task‐related OT (30 min/day) vs. bilateral robot training (30 min/day), plus routine rehab in both	18 × 30 min (540 min; 9 h)	12/12	FMA‐UE; Barthel/MBI	Multiarm: N; Hybrid: N
Wang et al. 2023 [71]	Subacute (mean onset ~ 90–102 days)	Soft glove: Soft robotic glove (name NR)	Usual care: conventional rehabilitation (PT + acupuncture + OT) without glove add‐on (glove arm received an additional 20 min/day)	14 × 20 min (280 min; 4.67 h)	23/23	RMT; Barthel/MBI	Multiarm: Y (3 arms; rTMS arm not used for robot vs. non‐robot synthesis); Hybrid: N

*Note*: This table summarizes randomized controlled trials eligible for the primary quantitative synthesis (robot‐assisted upper‐limb training vs. non‐robot comparators) at immediate post‐intervention. Trials in which both groups received robotics were not eligible for this primary contrast and were analyzed separately.

Abbreviations: EEG, electroencephalography; FMA‐UE, Fugl–Meyer assessment‐upper extremity; MBI, modified barthel index; MEP, motor‐evoked potential; NR, not reported; OT, occupational therapy; PT, physical therapy; RCT, randomized controlled trial; RMT, resting motor threshold; rTMS, repetitive transcranial magnetic stimulation.

### 3.2. Neuroplasticity Biomarker Families and Measurement Heterogeneity

Neuroplasticity outcomes were clustered into measures derived from TMS, electrophysiology (primarily EEG), and neuroimaging/hemodynamic methods (including fMRI, diffusion imaging, and fNIRS). Many studies operationalized neuroplasticity using constructs such as corticospinal excitability, interhemispheric balance, hemispheric engagement, and network‐level connectivity [26, 44, 80]. Biomarkers were frequently analyzed as secondary or exploratory endpoints, and prespecification of a single primary biomarker endpoint was uncommon; several studies reported multiple related features derived from the same acquisition. Measurement heterogeneity in selection, acquisition protocols, and analytic strategies limited comparability and constrained quantitative synthesis beyond the prespecified endpoints.

TMS‐based studies most often reported indices of corticospinal excitability and interhemispheric balance, including RMT, MEP amplitude or MEP presence, and inhibitory/facilitatory measures such as SICI or related paired‐pulse indices [26, 27, 71]. Some reports included additional markers such as motor mapping parameters or silent period measures and examined stimulation–training interactions or timing effects in hybrid protocols [46–48]. Reporting frequently emphasized within‐group change, and acquisition conditions varied (e.g., intensity selection, target muscles, and handling of absent responses). Details needed to interpret between‐group differences—such as whether results reflect change scores versus post‐intervention values and whether models adjusted for baseline—were inconsistently reported (NR). Prespecified handling of absent MEPs was uncommon, limiting the interpretability of amplitude‐based contrasts in cohorts with more severe impairment.

EEG studies described changes in oscillatory power, event‐related desynchronization/synchronization patterns during motor imagery or task performance, hemispheric symmetry indices (including rBSI and related measures), laterality indices, and connectivity metrics [29, 44, 70]. Several reports also included measures of aperiodic activity (e.g., spectral exponent indices), reflecting broader changes in cortical dynamics during rehabilitation [28, 54]. Diversity in preprocessing pipelines, frequency band definitions, channel/region selection, and statistical correction approaches constrained direct cross‐study comparison and typically precluded pooling. Tasks used to elicit sensorimotor activity varied (motor imagery versus movement execution or passive versus active paradigms), which is relevant because biomarker directionality and magnitude may depend on task demands, effort, and attention.

Neuroimaging and hemodynamic studies described changes in activation patterns, hemispheric engagement, and network connectivity using task‐based or resting‐state fMRI, diffusion tensor imaging, and fNIRS [55, 72, 80]. fNIRS studies commonly reported task‐related oxygenated hemoglobin responses and laterality indices, with some reports exploring resting‐state connectivity between motor and prefrontal regions; protocols differed in optode placement, tasks, and region selection [55, 60, 72–74]. MRI‐based studies examined activation shifts, resting‐state connectivity, and diffusion‐based indices related to corticospinal tract integrity, often relating these measures to the concurrent clinical status. Outcomes were frequently expressed as region‐based indices or exploratory network metrics, limiting the harmonization of endpoints across studies.

Exploratory linkage between biomarkers and behavioral outcomes was commonly evaluated using within‐study correlations of change scores or predictive modeling using baseline biomarker measures. Associations were reported between changes in corticospinal excitability or network measures and changes in upper‐limb motor scales or task performance [31, 39, 43, 45, 50, 51, 55, 62, 63, 75, 77, 78]. Reporting of correlation coefficients, model specifications, covariate adjustment, and multiplicity control was variable, and repeated‐measures designs aligned to clinically meaningful trajectories were uncommon. Most biomarker–behavior relationships were therefore reported as exploratory associations rather than as prespecified mechanistic tests.

### 3.3. Risk of Bias Patterns Across Designs

RCTs were assessed with RoB 2. Overall, 9 of 24 trials were judged at high risk of bias and 15 of 24 as raising some concerns. The most recurrent limitation concerned selection of the reported result, with some concerns in 23 trials and high risk in one trial. Additional concerns involved the randomization process and missing outcome data, whereas outcome measurement was more often rated as low‐risk. These patterns indicate that several randomized trials provided useful comparative evidence but that biomarker outcomes were often vulnerable to incomplete prospective specification and analytic flexibility.

**Figure 2:**
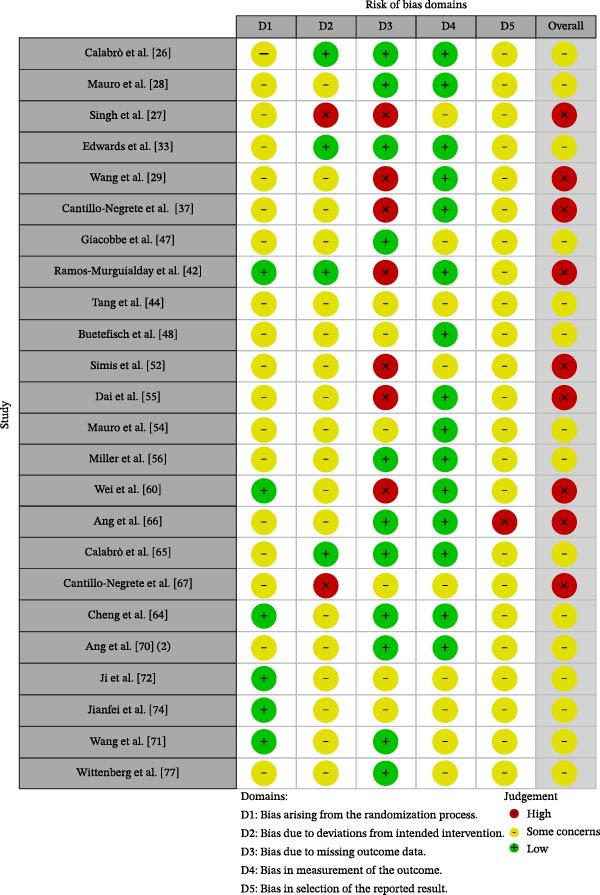
Risk of bias (RoB 2) for randomized trials.

**Figure 3:**
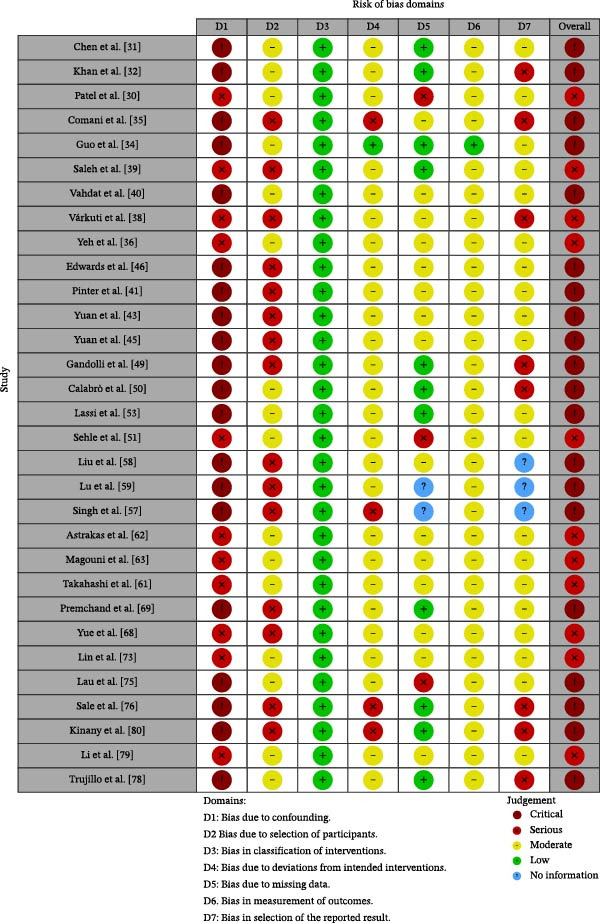
Risk of bias (ROBINS‐I) for nonrandomized studies.

Nonrandomized intervention studies were assessed with ROBINS‐I. Critical risk of bias was assigned to 20 of 31 studies and serious risk to 11 of 31 studies, with confounding as the dominant driver. Bias due to participant selection, deviations from intended interventions, and outcome measurement also contributed to several reports. For this reason, nonrandomized biomarker findings were treated as exploratory and hypothesis‐generating, particularly when neural changes were reported without concurrent control comparisons or in contexts where spontaneous recovery, background rehabilitation, or co‐interventions could plausibly explain observed changes. Summary risk‐of‐bias profiles are presented in Figure 2 for randomized trials and Figure 3 for nonrandomized studies.

### 3.4. Quantitative Synthesis of Prespecified Key Outcomes at Post‐Intervention

**Figure 4:**
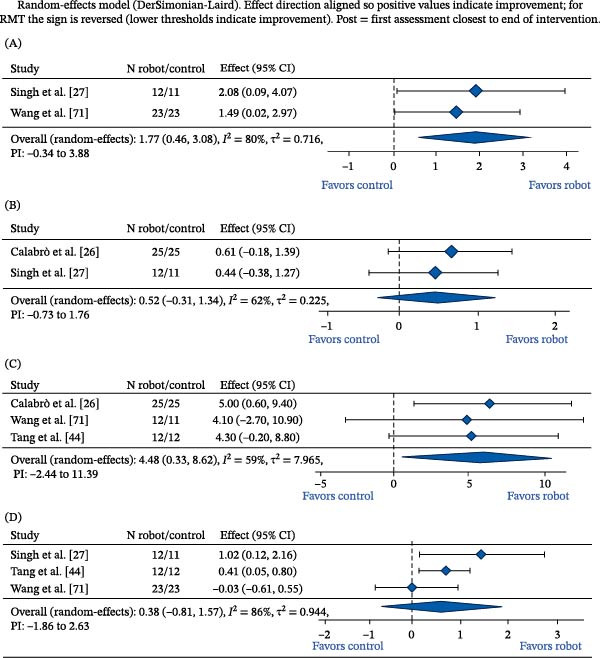
Forest plots of key outcomes at post‐intervention (robot‐assisted training vs. non‐robot control). (A) Ipsilesional RMT (SMD, Hedges’ *g*: change). (B) Ipsilesional MEP amplitude (SMD, Hedges’ *g*: post). (C) FMA‐UE total (MD: post). (D) Barthel index/modified Barthel index (SMD, Hedges’ *g*: post). ADL, activities of daily living; CI, confidence interval; DL, DerSimonian–Laird; FMA‐UE, Fugl–Meyer assessment‐upper extremity; *I*², inconsistency; MBI, modified Barthel index; MD, mean difference; MEP, motor‐evoked potential; PI, prediction interval; RMT, resting motor threshold; SMD, standardized mean difference; *τ*², between‐study variance.

Random‐effects models were used when at least two RCTs reported compatible outcomes for the primary robot‐versus‐non‐robot contrast at immediate post‐intervention. The pooled analyses were intentionally restricted to randomized evidence and to the primary contrast to avoid conflating robotics effects with adjunct, BCI‐guided, or robot‐only comparison effects. GRADE certainty ratings are reported in Table 2, pooled estimates are summarized in Table 3 and displayed in Figure 4, and sensitivity analyses for the key post‐intervention outcomes are reported in Supporting Information 4: Material S4.

**Table 2:** Summary of findings and GRADE (post): robot‐assisted training vs. non‐robot control.

Outcome (post)	Studies (*k*)/participants (*N*)	Effect [95% CI]	Heterogeneity (*I* ^2^; *τ* ^2^)	Prediction interval	Certainty (GRADE) + reasons
Ipsilesional RMT (primary mechanistic)	2/69	SMD (Hedges’ *g*) = 1.77 [0.46, 3.08]	*I* ^2^ = 80%; *τ* ^2^ = 0.716	[−0.34, 3.88]	Low (downgraded for inconsistency and imprecision)
Ipsilesional MEP amplitude (key secondary mechanistic)	2/73	SMD (Hedges’ *g*) = 0.52 [−0.31, 1.34]	*I* ^2^ = 62%; *τ* ^2^ = 0.225	[−0.73, 1.76]	Very low (downgraded for risk of bias, inconsistency, and imprecision)
FMA‐UE total (clinical anchor)	3/97	MD = 4.48 [0.33, 8.62]	*I* ^2^ = 59%; *τ* ^2^ = 7.965	[−2.44, 11.39]	Low (downgraded for inconsistency and imprecision)
Barthel index/modified Barthel index (ADL anchor)	3/93	SMD (Hedges’ *g*) = 0.38 [−0.81, 1.57]	*I* ^2^ = 86%; *τ* ^2^ = 0.944	[−1.86, 2.63]	Very low (downgraded for risk of bias, inconsistency, and imprecision)

*Note:* This summary of findings table reports pooled estimates and GRADE certainty for prespecified key outcomes at immediate post‐intervention (post), restricted to the primary contrast (robot‐assisted training vs. non‐robot control). Effect direction is aligned so positive values indicate improvement; for RMT the sign is reversed because lower thresholds indicate improvement.

Abbreviations: ADL, activities of daily living; CI, confidence interval; GRADE, grading of recommendations, assessment, development, and evaluation; *I* ^2^, *I*‐squared statistic; MBI, modified Barthel index; MD, mean difference; MEP, motor‐evoked potential; PI, prediction interval; RCT, randomized controlled trial; RMT, resting motor threshold; RoB, risk of bias; SD, standard deviation; SMD, standardized mean difference (Hedges’ *g*); *τ* ^2^, between‐study variance.

**Table 3:** Meta‐analysis overview (random‐effects and DerSimonian–Laird): robot‐assisted training vs. non‐robot control.

Key outcome	Timepoint	*k*	Total *N* (robot/control)	Effect metric	Value type	Pooled effect (95% CI)	*p*	*I* ^2^ (%)	Prediction interval (95%)
Ipsilesional RMT (primary mechanistic)	Post	2	69 (35/34)	SMD (Hedges’ *g*)	Change	1.77 (0.46 to 3.08)	0.008	80	−0.34 to 3.88
Ipsilesional MEP amplitude (key secondary mechanistic)	Post	2	73 (37/36)	SMD (Hedges’ *g*)	Post	0.52 (−0.31 to 1.34)	0.221	62	−0.73 to 1.76
FMA‐UE total (clinical anchor)	Post	3	97 (49/48)	MD	Post	4.48 (0.33 to 8.62)	0.034	59	−2.44 to 11.39
Barthel index/modified Barthel index (ADL anchor)	Post	3	93 (47/46)	SMD (Hedges’ *g*)	Post	0.38 (−0.81 to 1.57)	0.530	86	−1.86 to 2.63

*Note*: This table provides an overview of the primary random‐effects meta‐analyses at post for the primary robot versus non‐robot contrast, including effect metrics (MD or Hedges’ *g*), heterogeneity statistics, and prediction intervals when estimable.

Abbreviations: CI, confidence interval; DL, DerSimonian–Laird; FMA‐UE, Fugl–Meyer assessment‐upper extremity; *I* ^2^, *I*‐squared statistic; MBI, modified Barthel index; MD, mean difference; MEP, motor‐evoked potential; NA, not applicable; *p*, *p*‐value; PI, prediction interval; RCT, randomized controlled trial; RMT, resting motor threshold; SD, standard deviation; SE, standard error; SMD, standardized mean difference (Hedges’ *g*); *τ* ^2^, between‐study variance

Ipsilesional RMT was pooled from two trials including 69 participants. The pooled estimate favored robot‐assisted training (standardized MD [SMD] 1.77, 95% CI: 0.46 to 3.08), but heterogeneity was substantial, and the prediction interval crossed the null, indicating that the expected effect may vary across settings and protocols. The certainty of evidence was low because of inconsistency and imprecision.

The ipsilesional MEP amplitude was pooled from two trials including 73 participants. The pooled estimate was imprecise and compatible with no effect (SMD 0.52, 95% CI: −0.31 to 1.34), with moderate heterogeneity and a prediction interval crossing the null. The certainty of evidence was very low because of risk of bias, inconsistency, and imprecision.

Upper‐limb impairment measured with FMA‐UE was pooled from three trials, including 97 participants. The pooled MD favored robot‐assisted training (MD 4.48 points, 95% CI: 0.33–8.62), but heterogeneity was moderate and the prediction interval crossed the null. The certainty of evidence was low because of inconsistency and imprecision.

ADL, measured using the Barthel Index or Modified Barthel Index, were pooled from three trials including 93 participants. The pooled estimate was imprecise and compatible with no effect (SMD 0.38, 95% CI: −0.81 to 1.57), with considerable heterogeneity and a wide prediction interval. The certainty of evidence was very low because of risk of bias, inconsistency, and imprecision.

Across all pooled outcomes, the number of contributing trials was small (*k* = 2–3), prediction intervals crossed the null, and certainty ranged from low to very low. These findings support cautious interpretation of pooled point estimates and preclude strong mechanistic or clinical claims based on the current randomized evidence.

### 3.5. Evidence Beyond Pooled Outcomes and the Primary Contrast

**Figure 5:**
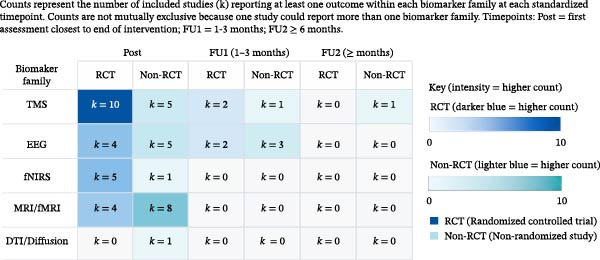
Evidence map of neuroplasticity biomarkers by design and timepoint. DTI, diffusion tensor imaging; EEG, electroencephalography; fMRI, functional magnetic resonance imaging; fNIRS, functional near‐infrared spectroscopy; FU, follow‐up; Post, post‐intervention; RCT, randomized controlled trial; TMS, transcranial magnetic stimulation.

Most biomarker outcomes could not be pooled because they were operationalized as multiple region‐, channel‐, or network‐level measures, because acquisition and analytic pipelines differed, or because reporting did not provide sufficient quantitative detail for compatible effect‐size computation. The distribution of biomarker evidence by study design and timepoint is summarized in Figure 5 [44, 55, 70, 72, 76, 80]. EEG outcomes were most frequently reported at immediate post‐intervention, with a smaller body extending to short follow‐up [44, 70, 76]. Imaging and hemodynamic outcomes clustered at post‐intervention, with limited representation beyond that point [55, 72]. Diffusion and fMRI studies were also concentrated at post‐intervention, and follow‐up imaging was uncommon [80]. The evidence map highlights the limited availability of longer‐term biomarker trajectories and the scarcity of multimodal designs aligning biomarker changes with sustained clinical outcomes.

Additional prespecified comparison classes were summarized at the individual study level rather than being pooled to avoid conflating distinct contrasts. Individual study effects for these contrasts are reported in Table 4 [47, 55, 74, 75]. These include comparisons where robotics served as a platform and the contrast isolated an adjunct or a guidance/contingency component (robot + adjunct versus robot‐only; neural guidance/BCI versus noncontingent/sham guidance with identical robotic practice), as well as designs with dependencies (e.g., crossover timing comparisons) that precluded independent pooling. Interpretation of these effects is therefore limited to incremental effects within robotic platforms rather than evidence for robotics versus non‐robot comparators.

**Table 4:** Additional prespecified comparison classes (individual study effects; no pooling).

Comparison class	Study	Outcome	*N* (Exp/control)	Effect metric	Effect (point estimate)	Notes
BCI or neural guidance vs. robot	Wang et al. [29]	FMA‐UE total (clinical anchor)	13/11	MD	−0.12	EEG/MI‐guided robotics vs. non‐contingent/sham guidance with robotics
Robot plus adjunct vs. robot only	Dai et al. [55]	FMA‐UE total (clinical anchor)	16/16	MD	7.96	Adjunct added to robotics vs. robot only
Robot plus adjunct vs. robot only	Giacobbe et al. [47]	Ipsilesional MEP amplitude (key secondary mechanistic)	5/2	SMD (Hedges’ *g*)	−0.35	Crossover; sham arm split across timing comparisons (not independent)
Robot plus adjunct vs. robot only	Giacobbe [47]	Ipsilesional MEP amplitude (key secondary mechanistic)	5/2	SMD (Hedges’ *g*)	−0.80	Crossover; sham arm split across timing comparisons (not independent)
Robot plus adjunct vs. robot only	Giacobbe [47]	Ipsilesional MEP amplitude (key secondary mechanistic)	6/2	SMD (Hedges’ *g*)	−0.29	Crossover; sham arm split across timing comparisons (not independent)
Robot plus adjunct vs. robot only	Song et al. [74]	Barthel index/modified Barthel index (ADL anchor)	25/25	SMD (Hedges’ *g*)	0.67	Adjunct added to robotics vs. robot only
Robot plus adjunct vs. robot only	Song et al. [74]	FMA‐UE total (clinical anchor)	25/25	MD	6.00	Adjunct added to robotics vs. robot only

*Note:* This table summarizes individual study effects for additional prespecified comparison classes (robot + adjunct vs. robot only; BCI/neural guidance vs. sham/noncontingent guidance with identical robotic practice). These comparisons are reported separately to avoid conflating robotics effects with adjunct or modality effects; pooling was not performed when insufficient compatible trials were available or when design dependencies precluded independent estimates.

Abbreviations: BCI, brain–computer interface; EEG, electroencephalography; FMA‐UE, Fugl–Meyer assessment‐upper extremity; iTBS, intermittent theta‐burst stimulation; MD, mean difference; MEP, motor‐evoked potential; RT, robot training; SMD, standardized mean difference (Hedges’ *g*); tDCS, transcranial direct current stimulation.

### 3.6. Safety and Tolerability

Safety reporting was heterogeneous. Thirty‐seven studies explicitly indicated that adverse events were not reported, whereas 18 studies provided some adverse event information. Reported events were typically mild and transient and included fatigue, headache, tingling, redness, and musculoskeletal or shoulder discomfort [47, 71]. One randomized trial reported a minor seizure that resolved without lasting sequelae and led to intervention discontinuation [70]. Incomplete and inconsistent adverse event reporting limited comparative assessment of tolerability across devices, stroke stages, and hybrid intervention components.

## 4. Discussion

### 4.1. What the Evidence Can Answer

This review addresses three related but distinct questions. The first is comparative clinical effectiveness: whether robot‐assisted upper‐limb training improves clinical outcomes compared with non‐robot rehabilitation. The second is mechanistic observation: whether robot‐assisted training is accompanied by measurable changes in neurophysiological or neuroimaging biomarkers. The third is mechanistic linkage: whether biomarker changes explain, predict, or mediate behavioral improvement.

The current evidence is strongest for cautious statements about impairment‐level change and weakest for causal mechanistic linkage. In the primary randomized contrast, robot‐assisted training was associated with improvement in FMA‐UE and with a pooled shift in ipsilesional RMT immediately post‐intervention. However, each pooled endpoint was based on only two or three trials, heterogeneity was moderate to substantial, prediction intervals crossed the null, and certainty ranged from low to very low. Therefore, the pooled estimates should be interpreted as preliminary comparative signals rather than definitive evidence of a consistent robotics‐induced neuroplastic mechanism.

The nonrandomized literature broadens the mechanistic map by describing EEG, fNIRS, fMRI, diffusion, and TMS findings across diverse robotic paradigms. Nevertheless, these studies are mainly useful for hypothesis generation. They do not establish that robotic training caused the observed biomarker changes, nor that such biomarker changes mediated clinical recovery. This distinction guided the synthesis and should frame the interpretation of the review.

### 4.2. Clinical Outcomes: Impairment Gains and Uncertain Translation to Daily Activities

The pooled estimate for FMA‐UE suggests that robot‐assisted training can improve upper‐limb impairment at the end of treatment compared with non‐robot comparators [26, 27, 44]. At the same time, heterogeneity and prediction intervals indicate that benefit is not uniform and is likely context‐dependent. Variation is plausible at several levels. Interventions differed in device class and target (hand‐focused versus more proximal platforms), assistance strategy, and total dose. Comparator intensity also differed: dose‐matched active therapy can reduce between‐group differences, whereas usual‐care comparators may allow larger incremental effects. Trial populations differed in stroke stage and severity, and baseline biological capacity for restitution is likely to influence responsiveness to any high‐dose motor practice.

Clinical significance also depends on the level at which recovery is assessed. Impairment scales capture change within a standardized examination framework, but improvements on impairment scales do not guarantee improved spontaneous use of the paretic limb or improved independence in daily activities. Evidence for translation to global ADL was weaker in the pooled results. Barthel/modified Barthel effects were highly heterogeneous and imprecise and were rated very low certainty [27, 44, 71]. Several nonexclusive explanations are plausible. Barthel‐type measures are not specific to upper‐limb dexterity and may have ceiling effects, limiting sensitivity to changes in hand function. Gains in impairment may not translate into independence if task practice does not address context‐specific activities, if learned strategies rely on assistance that does not generalize, or if other limiting impairments (e.g., balance, cognition, and aphasia) constrain function. Timing also matters: participation‐level changes may lag behind impairment gains, and immediate post‐intervention assessment may underestimate downstream functional effects when uptake into everyday behavior requires time and environmental support.

The imbalance between impairment gains and ADL uncertainty should not be interpreted as evidence that robotics is clinically irrelevant. Instead, it highlights that current trials provide limited and inconsistent support for participation‐level outcomes and that trial design must align intervention targets with the outcome selection. Studies that incorporate measures of functional task performance, real‐world arm use, and movement quality, alongside impairment scales and longer follow‐up, would strengthen interpretation and better match clinical priorities.

### 4.3. Interpreting Biomarker Changes: Measurement Variability and Task Mismatch

TMS outcomes were the only mechanistic endpoints eligible for pooling in the primary randomized contrast. The pooled RMT finding suggests that robot‐assisted training may be associated with a post‐intervention shift consistent with increased corticospinal excitability, but this estimate was based on only two trials, heterogeneity was substantial, and certainty remained low [27, 71]. The MEP amplitude was also based on two trials and showed an imprecise estimate compatible with no effect [26, 27]. These findings should therefore be interpreted cautiously. RMT and MEP amplitudes are sensitive to protocol choices, baseline corticospinal tract integrity, stimulation intensity, target muscle, pre‐activation state, and the handling of absent or near‐zero responses. In cohorts with more severe paresis, absent MEPs are particularly relevant, and different analytic decisions can change group estimates in ways that are difficult to interpret physiologically.

EEG, fNIRS, and MRI‐based studies suggest that robot‐assisted training can be accompanied by changes in distributed motor and cognitive control networks [29, 44, 55, 70, 72, 76, 80]. However, these modalities were reported using heterogeneous tasks, preprocessing pipelines, regions of interest, and outcome definitions. As a result, these findings are best interpreted as evidence of neural engagement during or after training rather than as directly comparable biomarkers of recovery.

The clinical meaning of a biomarker also depends on the measurement context. Resting measures may not capture the neural control strategies used during trained movements, whereas task‐based measures are more directly linked to performance but are more sensitive to effort, attention, fatigue, task design, and analysis choices. Future trials should therefore prespecify biomarker acquisition and analysis pipelines, align neural measures with trained behaviors, and report how missing or absent responses, especially absent MEPs, were handled.

### 4.4. Recovery Versus Compensation and Limits to Biomarker–Behavior Linkage

Neurodynamic change should not be equated automatically with behavioral restitution. After stroke, motor improvement can reflect restitution of impaired motor control, compensation through alternative movement strategies, or both. This distinction is important for robotic rehabilitation because device constraints, assistance levels, feedback, and task goals can shape how movement success is achieved. Without concurrent kinematic or movement‐quality measures, it is difficult to determine whether a neural change reflects restoration of premorbid control, adaptive compensation, nonspecific training exposure, or concurrent rehabilitation effects.

Biomarker–behavior relationships were frequently reported as correlations or exploratory prediction models. These analyses are useful for generating hypotheses about responsiveness and biological capacity for recovery, but they do not establish mediation or causality. Stronger mechanistic inference would require trials showing that the intervention changes the biomarker, that biomarker change precedes or predicts behavioral change, and that the relationship remains robust after accounting for baseline severity, dose, comparator intensity, and other plausible confounders.

### 4.5. Heterogeneity, Attribution, and Hybrid Robotic Paradigms

The heterogeneity observed in pooled outcomes and in the wider biomarker literature likely reflects variation at several levels. Patient factors include stroke stage, severity, and biological capacity for restitution, which influence both responsiveness to training and the feasibility and stability of certain biomarkers. Intervention factors include device class, training targets (proximal reaching versus distal hand function), assistance strategy, feedback modalities, and total dose. Comparator factors include intensity and content of non‐robot therapy and the extent to which co‐interventions are balanced or documented. These sources of variation are not merely statistical noise; they represent plausible effect modifiers that should inform future trial design and stratified analyses. The current evidence base, however, is too sparse in randomized mechanistic outcomes to support robust subgroup conclusions.

Hybrid designs add an additional layer of attribution complexity. Randomized studies that compare robot‐plus‐adjunct approaches with robot‐only practice, or compare brain‐guided/neural‐contingent control with noncontingent or sham guidance delivered through the same robotic device, estimate incremental effects on top of robotics rather than the effect of robotics versus non‐robot rehabilitation [29, 47, 55, 74, 75]. These effects are therefore most appropriately interpreted as evidence about adjuncts or about the added value of contingency within a robotic platform, not as evidence for robotics per se. The broader implication is that mechanistic claims should remain aligned with the comparison that was actually tested.

### 4.6. Implications for Practice and Priorities for Research

For clinical practice, the current evidence supports robot‐assisted upper‐limb rehabilitation as a structured way to deliver repeated, task‐oriented practice after stroke. The most defensible clinical conclusion is at the impairment level: robot‐assisted training may improve FMA‐UE scores at the end of treatment compared with non‐robot comparators. Evidence for ADL remains uncertain, and current biomarker findings should not be used as routine surrogate markers of functional recovery.

Clinically, robotic therapy is likely useful when it is integrated into a broader rehabilitation program rather than delivered as an isolated technology. Programs should define patient‐specific goals, document dose and comparator therapy, monitor movement quality, and combine device‐supported repetition with task‐specific practice relevant to daily life. Patient selection, stroke stage, baseline severity, residual corticospinal integrity, cognition, and capacity for active participation may all influence the response.

For research, future trials should prioritize adequately powered randomized designs with active dose‐matched comparators, harmonized biomarker protocols, transparent reporting of co‐interventions, and longitudinal follow‐up. Mechanistic endpoints should be prespecified and linked to movement quality and functional outcomes. Biomarker analyses should distinguish exploratory associations from clinically meaningful effects and should avoid treating neural changes as surrogate endpoints unless this relationship is directly tested.

### 4.7. Strengths and Limitations

This review has several methodological strengths. The work followed PRISMA 2020 guidance, with transparent reporting of eligibility criteria, screening, and synthesis decisions [17]. The search strategy covered multiple major databases and was conducted without time restrictions, reducing the likelihood of missing foundational mechanistic studies. Study selection and data extraction were performed independently by two reviewers, with substantial interrater agreement (Cohen’s kappa 0.71 for title/abstract screening and 0.75 for full‐text assessment), and discrepancies were resolved by consensus with adjudication when needed. Risk of bias was evaluated using validated tools tailored to study design (RoB 2 for randomized trials and ROBINS‐I for nonrandomized intervention studies), supporting a structured appraisal of internal validity [18, 19]. The certainty of evidence for prespecified key outcomes was rated using GRADE and summarized in a dedicated Summary of Findings table, which helps align conclusions with evidential strength and uncertainty [24].

Several design choices were made to improve interpretability and reduce over‐attribution. Comparison classes were prespecified, and the meta‐analysis was restricted to the primary contrast (robot‐assisted training versus non‐robot comparators) at the first post‐intervention assessment, while adjunct‐on‐robot and guidance/contingency comparisons were synthesized separately. This approach limits conflation of robotics effects with incremental effects of co‐interventions or platform‐specific guidance. Random‐effects models were used to account for expected clinical and methodological variability, and sensitivity analyses were planned to examine robustness under alternative assumptions. When meta‐analysis was not appropriate, narrative synthesis followed SWiM guidance, and studies were grouped by biomarker family and design, with an evidence map used to clarify where evidence is concentrated or sparse [23].

Important limitations reflect both the nature of the primary literature and practical constraints of synthesis. The evidence base is heterogeneous with respect to robot class, control strategy, treatment dose, comparator intensity, stroke stage, and baseline severity. Such diversity reduces the interpretability of pooled point estimates as a single “robotics effect” and is consistent with the wide prediction intervals observed for several outcomes. Pooling was feasible for only a small subset of outcomes, and the number of contributing randomized trials was limited for key mechanistic endpoints (*k* = 2 for ipsilesional RMT and MEP amplitude) and for clinical anchors (*k* = 3 for FMA‐UE and Barthel/modified Barthel), increasing imprecision and limiting exploration of effect modifiers. Formal assessment of small‐study effects and publication bias was not possible because each pooled analysis included fewer than 10 studies, and subgroup/meta‐regression analyses were not supported by the available data.

Limitations are particularly salient for mechanistic inference. Biomarker definitions and acquisition protocols varied widely (e.g., TMS stimulation parameters and handling of absent MEPs; EEG preprocessing and feature selection; imaging tasks, regions of interest, and connectivity metrics). This heterogeneity, together with selective reporting concerns identified in RoB 2, constrains cross‐study comparability and increases the risk that nominally consistent “directional” biomarker findings may reflect analytic flexibility rather than a shared physiological signal. Nonrandomized biomarker studies were frequently judged to be at serious or critical risk of bias due to confounding, and results from these designs should be interpreted as hypothesis‐generating rather than causal. Sparse follow‐up biomarker data further limit conclusions regarding the durability of neurodynamic changes and their temporal relationship with functional recovery.

Finally, restricting inclusion to English‐language full texts may introduce language bias, although it also reduces the risk of misclassification of complex neurophysiological methods and endpoints. The protocol was registered and updated with a date‐stamped audit trail; however, some clarifications were made after initial registration, which may limit strict prospective interpretation, even though core eligibility criteria and prespecified key outcomes for quantitative synthesis were maintained. Overall, the conclusions are necessarily cautious and are framed to reflect low‐ to very low certainty for several key outcomes and the current limitations of mechanistic trial evidence.

The most important limitation for interpretation is the small randomized evidence base available for each pooled endpoint. Although the review identified 55 eligible studies overall, the primary meta‐analyses were based on only four RCTs, and each pooled outcome included only two or three trials. Consequently, pooled point estimates should not be interpreted as stable estimates of a general robotics effect. They are better understood as preliminary estimates within a heterogeneous and still‐developing mechanistic evidence base.

## 5. Conclusion

Robot‐assisted upper‐limb rehabilitation after stroke may improve impairment‐level outcomes at the end of treatment compared with non‐robot comparators, but the randomized evidence remains limited. Each pooled endpoint was based on only two or three trials, heterogeneity was moderate to substantial, prediction intervals crossed the null, and certainty of evidence ranged from low to very low. Effects on ADL remain uncertain.

Neuroplasticity biomarkers suggest that robot‐assisted training can be accompanied by changes in corticospinal excitability and distributed neural network activity, but current evidence does not establish that these changes are causal, durable, or clinically meaningful surrogates of recovery. Biomarker findings should therefore be interpreted as exploratory mechanistic evidence rather than proof of cortical restitution. Future trials should use rigorous dose‐matched comparators, harmonized biomarker protocols, movement quality metrics, and longitudinal designs that explicitly test biomarker–outcome relationships.

## Author Contributions

Conceptualization: Rocco Salvatore Calabrò, Andrea Calderone, and Angelo Quartarone. Methodology: Rocco Salvatore Calabrò, Andrea Calderone, Alessandro Marco De Nunzio, Serena Filoni, and Angelo Quartarone. Software, formal analysis: Andrea Calderone and Alessandro Marco De Nunzio. Validation: Rocco Salvatore Calabrò, Andrea Calderone, and Serena Filoni. Investigation: Rocco Salvatore Calabrò, Andrea Calderone, Lilla Bonanno, Antonino Naro, Alessandro Marco De Nunzio, and Serena Filoni. Resources: Rocco Salvatore Calabrò, Antonino Naro, and Angelo Quartarone. Data curation, visualization: Andrea Calderone, Lilla Bonanno, and Alessandro Marco De Nunzio. Writing – original draft preparation: Andrea Calderone, Rocco Salvatore Calabrò, and Alessandro Marco De Nunzio. Writing – review and editing: Rocco Salvatore Calabrò, Andrea Calderone, Lilla Bonanno, Antonino Naro, Alessandro Marco De Nunzio, Serena Filoni, and Angelo Quartarone. Supervision, project administration, funding acquisition: Rocco Salvatore Calabrò and Angelo Quartarone.

## Funding

This study was supported by the Current Research Funds 2026, Ministry of Health, Italy.

## Disclosure

All authors have read and agreed to the published version of the manuscript.

## Ethics Statement

As this systematic review involves secondary data analysis from previously published studies, no new ethical approval was required.

## Consent

The authors have nothing to report.

## Conflicts of Interest

The authors declare no conflicts of interest.

## Supporting Information

Additional supporting information can be found online in the Supporting Information section.

## Supporting information


**Supporting Information 1** PRISMA 2020 checklist.

**Supporting Information 2** Appendix S1: Methods and transparency tables, including full search strategies and decision rules.

**Supporting Information 3** Table S3: Summary of included studies.

**Supporting Information 4** Table S4: Sensitivity analyses for key outcomes at post‐intervention.

## Data Availability

All data analyzed in this systematic review were extracted from previously published studies. The extracted data and analytic decisions are summarized in the manuscript and Supporting Information section.
