# Supplementary material for: Robot‐Assisted Upper‐Limb Rehabilitation After Stroke: A Systematic Review and Meta‐Analysis of Cortical Reorganization and Neuroplasticity Biomarkers
Source: Neural Plast. 2026 Jun 5;2026:9282578. doi: 10.1155/np/9282578 (PMC13238253; doi:10.1155/np/9282578)
Supplement: Supplementary file 2 — Supporting Information 2 Appendix S1: Methods and transparency tables, including full search strategies and decision rules. [file NP-2026-9282578-s001.docx]

**SUPPLEMENTARY MATERIAL 2 (Appendix S1): Methods and transparency tables**

This appendix provides structured, reproducible decision rules and operational definitions underpinning the review methods.

**List of tables**

Table S2A. Review planning, protocol record, reporting standard, and key dates

Table S2B. PICO operational definitions

Table S2C. Eligibility criteria (inclusion/exclusion) and decision rules

Table S2D. Search strategy dossier (Part 1: sources and approach; Part 2: full core search strings)

Table S2E. Study selection workflow and quality control

Table S2F. Data extraction framework and outcome operationalization (Panel A: extraction fields; Panel B: outcome hierarchy and selection rules)

Table S2G. Timepoint harmonization and value-type rules

Table S2H. Statistical synthesis decision rules

Table S2I. Missing data handling and imputation log rules

Table S2J. Risk of bias, certainty of evidence, and publication bias plan

Table S2K. Reproducibility checklist (computational environment and analytic transparency)

**Table S2A. Review planning, protocol record, reporting standard, and key dates**

Purpose: Provide an auditable record of protocol registration, reporting standards, and prespecified methodological anchors.

| **Item** | **Specification** | **Source/Notes** |
| --- | --- | --- |
| Review type | Systematic review and meta-analysis of neuroplasticity biomarkers in robot-assisted upper-limb rehabilitation after stroke. | Manuscript Methods. |
| Protocol registration | PROSPERO registration with versioned updates (see notes). | Registered in PROSPERO on 18 January 2025; Version 2.0 published 10 January 2026 (CRD42025635558). Planned administrative update dated 1 February 2026 to align wording with the final manuscript; core eligibility and key outcomes unchanged. |
| Reporting guideline | PRISMA 2020. | Methods; citation [17]. |
| Databases searched | PubMed; Web of Science; Embase; Cochrane Library; EBSCOhost; Scopus. | Final searches executed 12 January 2026. |
| Search coverage | From inception through 31 December 2025; no time restrictions applied. | Controlled vocabulary (MeSH/Emtree) plus free-text keywords. |
| Population | Adults (>=18 years) with ischemic or hemorrhagic stroke and upper-limb motor impairment. | PICO/Eligibility; PROSPERO Population. |
| Interventions | Robot-assisted upper-limb rehabilitation via end-effector robots, exoskeletons, wearable hand devices, or soft robotic gloves. | PICO/Eligibility; PROSPERO Interventions. |
| Comparators | Dose-matched or usual-care non-robotic rehabilitation; sham/minimal intervention; alternative active interventions; and single-group pre-post designs when eligible. | PICO/Eligibility; PROSPERO Comparators/Study design. |
| Study designs | Randomized controlled trials; non-randomized controlled intervention studies; and single-group pre-post intervention studies (including feasibility/pilot studies when extractable). | PROSPERO Study design; Methods scope. |
| Primary outcomes | Neuroplasticity biomarkers; prespecified key outcomes for pooling: ipsilesional RMT (primary) and ipsilesional MEP amplitude (key secondary) at immediate post-intervention. | Outcome hierarchy in Methods. |
| Secondary outcomes | Clinical anchors: FMA-UE total and Barthel/Modified Barthel at post when available; additional validated upper-limb/activity measures, kinematic metrics, adherence, and adverse events extracted when reported. | Methods and PROSPERO Additional outcomes. |
| Timepoint harmonization | Post = first assessment closest to end of intervention; FU1 = 1-3 months; FU2 = >=6 months. | Methods; PROSPERO synthesis plan. |
| Risk of bias tools | RoB 2 for randomized trials; ROBINS-I for non-randomized intervention studies. | Methods; citations [18,19]. |
| Quantitative synthesis default | Random-effects meta-analysis when >=2 compatible randomized trials. | Methods; citation [20]. |
| Publication bias plan | Funnel plots and Egger regression when >=10 studies contribute to an outcome. | Methods; citation [25]. |
| Review timeline | Start date 11 January 2026; end date 28 February 2026. | PROSPERO timeline. |
| Funding source | Current Research Funds 2026, Ministry of Health, Italy (non-commercial). | PROSPERO funding. |

Legends: ADL, activities of daily living; EEG, electroencephalography; fMRI, functional magnetic resonance imaging; fNIRS, functional near-infrared spectroscopy; FMA-UE, Fugl-Meyer Assessment for Upper Extremity; FU1, follow-up 1 (1-3 months); FU2, follow-up 2 (>=6 months); MeSH, Medical Subject Headings; MEP, motor evoked potential; MBI, Modified Barthel Index; PRISMA, Preferred Reporting Items for Systematic Reviews and Meta-Analyses; PROSPERO, International Prospective Register of Systematic Reviews; RCT, randomized controlled trial; RMT, resting motor threshold; RoB 2, Risk of Bias 2; ROBINS-I, Risk Of Bias In Non-randomized Studies - of Interventions; TMS, transcranial magnetic stimulation.

**Table S2B. PICO operational definitions**

Purpose: Define the review question components and key operational decisions (including hybrid interventions).

| **PICO element** | **Operational definition** | **Decision rules / notes** |
| --- | --- | --- |
| Population | Adults aged >=18 years with ischemic or hemorrhagic stroke and upper-limb motor impairment. | Subacute and chronic stages eligible when reported; mixed diagnoses excluded unless stroke-only data are separable or obtainable from authors. |
| Intervention | Robot-assisted upper-limb rehabilitation delivered as a therapeutic component intended to improve motor performance. | Eligible device classes: end-effector robots; exoskeletons; wearable hand devices; soft robotic gloves. Control strategies not restricted (e.g., assist-as-needed, impedance/admittance, haptic guidance, resistance, perturbations, error augmentation) but extracted for subgroup/sensitivity analyses when reported. |
| Comparator | Usual care, conventional upper-limb rehabilitation, dose-matched non-robotic therapy, sham/minimal-intensity interventions, alternative active treatments, or no comparator in single-group pre-post studies. | Comparator content/intensity extracted and considered in planned subgroup/sensitivity analyses; no comparator-based exclusion criteria applied. |
| Outcomes (neuroplasticity) | At least one neurophysiological or neuroimaging outcome consistent with neuroplastic change (TMS, EEG, neuroimaging). | Operationalized as measurable change in neural activity, network organization, corticospinal function, or structural connectivity. |
| Outcome modalities | TMS metrics; EEG features; neuroimaging outcomes from fMRI, fNIRS, or diffusion-based structural imaging. | Peripheral physiology without explicit neural interpretation is not eligible. |
| Hybrid interventions | Protocols combining robotics with VR, BCI control, or neurofeedback. | Eligible only if robot-assisted training remains the primary therapeutic driver by dose and session structure; excluding hybrids prespecified for sensitivity analyses. |

Legends: BCI, brain-computer interface; EEG, electroencephalography; fMRI, functional magnetic resonance imaging; fNIRS, functional near-infrared spectroscopy; TMS, transcranial magnetic stimulation; VR, virtual reality.

**Table S2C. Eligibility criteria (inclusion/exclusion) and decision rules**

Purpose: Specify eligibility rules and adjudication considerations for borderline cases.

| **Domain** | **Include** | **Exclude** | **Operational decision rules / borderline cases** |
| --- | --- | --- | --- |
| Participants | Adults with stroke (ischemic or hemorrhagic) and upper-limb motor impairment; subacute and chronic stages. | Participants <18 years; non-stroke populations; mixed neurological diagnoses unless stroke-only data are separable or obtainable from authors. | Age threshold: >=18 years. If mixed diagnoses are reported, include only if stroke-only data are extractable or can be obtained from authors. |
| Intervention intent | Robot-assisted upper-limb rehabilitation delivered as therapeutic training intended to improve motor performance. | Robotic device used solely as an assessment tool with no therapeutic training program. | If device delivers the core movement practice and dosage -> eligible. |
| Body region | Upper-limb/upper-extremity training. | Lower-limb-only robotics. | Mixed upper- and lower-limb programs: include only if upper-limb robotic component and outcomes are clearly separable (otherwise NR and adjudicate). |
| Robot modality | End-effector, exoskeleton, wearable hand device, or soft robotic glove. | NR. | Robot classification extracted as reported; ambiguous devices: record as NR and document in extraction notes. |
| Comparators | Usual care, conventional therapy, dose-matched non-robotic therapy, sham/minimal interventions, alternative active treatments, or no comparator in single-group pre-post designs. | NR. | Comparator type and intensity recorded; multi-arm handled per synthesis rules. |
| Study design | RCTs; non-randomized controlled intervention studies; single-group pre-post intervention studies (including feasibility/pilot studies when biomarker data are extractable). | Case reports; cross-sectional studies without an intervention; non-interventional observational designs without a defined robotic intervention and without intervention-linked pre-post biomarker outcomes. | Observational designs eligible only when a defined robot-assisted intervention is delivered and pre-post biomarkers have a clear temporal link to the intervention period. |
| Outcomes (core eligibility) | At least one neuroplasticity outcome from TMS/EEG/neuroimaging with explicit neural interpretation. | Absent neuroplasticity outcomes or outcomes limited to peripheral physiology without explicit neural interpretation. | Neuroplasticity defined as measurable change in neural activity, network organization, corticospinal function, or structural connectivity. |
| Publication type | Original full-text clinical studies with extractable results. | Narrative/systematic reviews, meta-analyses, guidelines, editorials, letters, commentaries, protocols without results; conference abstracts without full methods and extractable data. | If a proceeding includes full methods and extractable results, adjudicate case-by-case; default exclusion if insufficient reporting. |
| Language | Full-text articles published in English. | Non-English full-text articles. | Rationale: consistent interpretation of biomarker definitions/analytic choices and reproducible extraction. |
| Hybrid interventions | Hybrid robotics + VR/BCI/neurofeedback when robotics delivers the core movement practice and dosage. | Hybrid studies where attribution to robotics is not feasible or robotics is not the primary therapeutic component. | Excluding hybrids prespecified for sensitivity analyses. |

Legends: BCI, brain-computer interface; EEG, electroencephalography; fMRI, functional magnetic resonance imaging; fNIRS, functional near-infrared spectroscopy; NR, not reported; TMS, transcranial magnetic stimulation; VR, virtual reality.

**Table S2D. Search strategy dossier (Part 1: sources and approach)**

Purpose: Document information sources, coverage, controlled vocabulary approach, and related procedures.

| **Component** | **Specification** | **Notes** |
| --- | --- | --- |
| Databases | PubMed; Web of Science; Embase; Cochrane Library; EBSCOhost; Scopus. | Final database searches executed 12 January 2026. |
| Coverage window | From inception through 31 December 2025. | No time restrictions applied. |
| Search concepts | Stroke AND upper-limb robotics AND neuroplasticity modality terms (TMS/EEG; fMRI/diffusion; fNIRS). | Three core strings used and adapted per database. |
| Controlled vocabulary | Database-specific controlled vocabulary terms (MeSH and Emtree) adapted per database. | Specific headings/field tags per database: NR. |
| Free-text terms | Supplemented with free-text keywords. | NR on exact field tags. |
| Additional methods | Reference list screening and forward citation tracking. | Used to identify additional eligible studies. |

Legends: DTI, diffusion tensor imaging; EEG, electroencephalography; Emtree, Embase subject headings; fMRI, functional magnetic resonance imaging; fNIRS, functional near-infrared spectroscopy; MeSH, Medical Subject Headings; NR, not reported; TMS, transcranial magnetic stimulation.

**Table S2D. Search strategy dossier (Part 2: full core search strings)**

Purpose: Provide the three full core search strings as developed (and adapted per database).

| **Core search string** | **Full search string (as developed; adapted per database)** |
| --- | --- |
| Search string 1 (TMS/EEG) | (stroke OR poststroke OR cerebrovascular accident OR cerebrovascular OR brain ischemia OR intracranial hemorrhage) AND (robot OR robotic OR robotics OR robot-assisted OR robot assisted OR rehabilitation robotics OR rehabilitation robot OR exoskeleton OR end-effector OR wearable robot OR robotic glove OR soft robotics) AND (transcranial magnetic stimulation OR motor evoked potential OR corticospinal excitability OR electroencephalography OR EEG) AND (upper limb OR upper extremity OR arm OR hand OR wrist). |
| Search string 2 (fMRI/diffusion) | (stroke OR poststroke OR cerebrovascular accident OR cerebrovascular OR brain ischemia OR intracranial hemorrhage) AND (robot OR robotic OR robotics OR robot-assisted OR robot assisted OR rehabilitation robotics OR rehabilitation robot OR exoskeleton OR end-effector OR wearable robot OR robotic glove OR soft robotics) AND (functional magnetic resonance imaging OR fMRI OR resting state fMRI OR resting-state fMRI OR diffusion tensor imaging OR DTI OR tractography) AND (upper limb OR upper extremity OR arm OR hand OR wrist). |
| Search string 3 (fNIRS) | (stroke OR poststroke OR cerebrovascular OR hemiparesis OR hemiplegia) AND (robot OR robotic OR robotics OR robot-assisted OR robot assisted OR exoskeleton OR end-effector OR "rehabilitation robot") AND ("near infrared spectroscopy" OR "near-infrared spectroscopy" OR NIRS OR fNIRS OR "functional near infrared spectroscopy" OR "functional near-infrared spectroscopy" OR "optical topography") AND (upper limb OR upper extremity OR arm OR hand OR wrist). |

Legends: DTI, diffusion tensor imaging; EEG, electroencephalography; fMRI, functional magnetic resonance imaging; fNIRS, functional near-infrared spectroscopy; NIRS, near-infrared spectroscopy; TMS, transcranial magnetic stimulation.

**Table S2E. Study selection workflow and quality control**

Purpose: Describe screening workflow, reviewer roles, agreement metrics, and documentation plan.

| **Stage** | **Process description** | **Personnel** | **QC metrics / decision rules** |
| --- | --- | --- | --- |
| Record management | Record management steps prior to screening (e.g., deduplication and record tracking). | Two reviewers: RSC and AC. | Records were merged and deduplicated using automated exact/near-duplicate detection (title/author/year) followed by manual verification in [Microsoft Excel]. |
| Title/abstract screening | Independent screening of titles and abstracts using predefined eligibility criteria. | Two reviewers: RSC and AC. | Discrepancies resolved by consensus; Cohen's kappa = 0.71. |
| Full-text assessment | Independent full-text screening for eligibility. | Two reviewers: RSC and AC. | Discrepancies resolved by consensus; third reviewer SF adjudicated when needed; Cohen's kappa = 0.75. |
| Documentation | PRISMA flow diagram used to report the selection process. | Two reviewers: RSC and AC. | Numerical PRISMA counts are reported in Results. |
| Additional searching | Reference list screening and forward citation tracking of included and relevant reports. | Two reviewers: RSC and AC. | Eligibility assessed using the same criteria as database records (implied). |

Legends: NR, not reported; PRISMA, Preferred Reporting Items for Systematic Reviews and Meta-Analyses; QC, quality control.

**Table S2F. Data extraction framework and outcome operationalization (Panel A: extraction fields)**

Purpose: List extracted variables and operational notes for data extraction.

| **Extraction domain** | **Items captured** | **Operational notes** |
| --- | --- | --- |
| Study design | Design category (randomized vs non-randomized controlled vs single-group pre-post; observational only if intervention-linked pre-post biomarkers). | Extracted using a standardized form; two reviewers extracted independently with cross-checking. |
| Participants | Participant characteristics; baseline documentation of upper-limb impairment; stroke diagnosis confirmation. | Details extracted as reported; mixed diagnoses included only if stroke-only data are separable/obtainable. |
| Stroke descriptors | Stroke stage (subacute/chronic) and severity when reported. | Recorded as reported; used for prespecified subgrouping when feasible. |
| Intervention | Intervention content; robot class; control strategy; training dose; session structure; hybrid vs standalone features. | Control strategies (assist-as-needed, impedance/admittance, etc.) extracted when reported. |
| Comparator | Comparator characteristics (usual care/conventional therapy/dose-matched/sham/minimal/active alternative) and dose/intensity when reported. | Comparator intensity and dosing extracted for interpretability and subgroup/sensitivity analyses. |
| Biomarker acquisition | Key biomarker acquisition details needed to judge compatibility (task, metric definition, timepoint, units/scale). | Extracted as reported; used to assess pooling eligibility. |
| Outcomes | All neuroplasticity outcomes and primary clinical outcomes; additional clinical, kinematic, adherence, and adverse event data when available. | Authors contacted when outcome data were incomplete or unclear. |

Legends: NR, not reported.

**Table S2F. Data extraction framework and outcome operationalization (Panel B: outcome hierarchy and selection rules)**

Purpose: Specify candidate measures and prespecified prioritization/selection rules by outcome family.

| **Outcome family** | **Candidate measures** | **Prespecified prioritization / selection rule** | **Notes** |
| --- | --- | --- | --- |
| TMS (key mechanistic) | RMT; active motor threshold; MEP amplitude; recruitment curve parameters; intracortical inhibition/facilitation. | Primary mechanistic outcome: ipsilesional RMT at immediate post-intervention. When both resting and active motor threshold were reported, prioritize RMT. Key secondary mechanistic outcome: ipsilesional MEP amplitude at immediate post-intervention, using unconditioned single-pulse MEP amplitude where possible. When multiple muscles or stimulation conditions were reported, extract the trial-designated primary measure; if not specified, prioritize a hand intrinsic target (e.g., FDI/APB) and the most commonly reported condition across trials, selecting the timepoint/condition with the most complete data. | Other TMS outcomes extracted when reported; pooling contingent on compatibility across trials. |
| EEG | Task-related spectral power; coherence/connectivity; laterality indices; event-related measures relevant to motor tasks. | Extract measures as reported; quantitative pooling only when outcomes are compatible across trials. | Motor-task relevance required per trial context. |
| Neuroimaging | fMRI activation/connectivity; fNIRS hemodynamic activation metrics; diffusion-based measures related to corticospinal pathway involvement. | Extract measures as reported; quantitative pooling only when outcomes are compatible across trials. | Specific acquisition/processing pipelines: NR. |
| Clinical (secondary) | FMA-UE total; Barthel Index/Modified Barthel Index; other validated upper-limb/activity scales (e.g., ARAT, WMFT) when reported. | Clinical anchors for key synthesis: FMA-UE total and Barthel/Modified Barthel at immediate post-intervention when available; additional scales extracted to support narrative synthesis and clinical interpretation. | ADL scales, kinematic metrics, adherence, and adverse events extracted when available. |

Legends: ADL, activities of daily living; APB, abductor pollicis brevis; ARAT, Action Research Arm Test; EEG, electroencephalography; FDI, first dorsal interosseous; fMRI, functional magnetic resonance imaging; fNIRS, functional near-infrared spectroscopy; FMA-UE, Fugl-Meyer Assessment for Upper Extremity; MEP, motor evoked potential; MBI, Modified Barthel Index; NR, not reported; RMT, resting motor threshold; TMS, transcranial magnetic stimulation; WMFT, Wolf Motor Function Test.

**Table S2G. Timepoint harmonization and value-type rules**

Purpose: Define harmonized timepoints and rules for choosing change scores versus post values.

| **Rule set** | **Operational definition** | **Implementation notes** |
| --- | --- | --- |
| Immediate post-intervention (post) | Defined as the first assessment conducted closest to the end of the intervention. | Used as the primary timepoint for key outcomes. |
| Follow-up 1 (FU1) | Follow-up assessments grouped as 1-3 months. | Synthesized separately when >=2 trials contributed. |
| Follow-up 2 (FU2) | Follow-up assessments grouped as >=6 months. | Synthesized separately when >=2 trials contributed. |
| Value type preference | Change scores were extracted preferentially when reported. | If change scores unavailable, post-intervention values were extracted. |
| Baseline imbalance handling | Baseline imbalance examined descriptively. | When meaningful imbalance suspected, sensitivity analyses compared post-score and change-score-based estimates when both computable. |
| Pre-post correlation | Pre-post correlations imputed when required for change-score variance. | Primary r = 0.50; sensitivity range r = 0.25 to 0.75. |

Legends: FU1, follow-up 1 (1-3 months); FU2, follow-up 2 (>=6 months); NR, not reported.

**Table S2H. Statistical synthesis decision rules**

Purpose: Specify pooling eligibility, model choice, effect metrics, directionality, heterogeneity reporting, and planned subgroup/sensitivity analyses.

| **Component** | **Decision rule (as prespecified)** | **Notes / NR** |
| --- | --- | --- |
| Eligibility for meta-analysis | Pool when at least two randomized trials report compatible neuroplasticity outcomes derived from comparable tasks, timepoints, and metrics. | Compatibility adjudication criteria beyond this statement: NR. |
| Default model | Random-effects models used as default to reflect clinical and methodological heterogeneity [20]. | DerSimonian-Laird tau^2 estimator with inverse-variance weighting; 95% CIs computed using the normal approximation (Wald-type). |
| Fixed-effect model | Compared with random-effects in sensitivity analyses. | Inverse-variance fixed-effect model. |
| Effect size metric | Use MD when outcomes are on the same directly comparable scale/unit. Use Hedges' g (SMD) when scales/units differ or acquisition conditions vary materially [21]. | Small-sample correction inherent to Hedges' g; computation details: NR. |
| Primary quantitative focus | Immediate post-intervention synthesis for key outcomes: ipsilesional RMT (primary) and ipsilesional MEP amplitude (key secondary). | Secondary syntheses for FMA-UE and Barthel/Modified Barthel when >=2 RCTs contribute compatible data at the same harmonized timepoint. |
| Follow-up syntheses | FU1 and FU2 analyzed separately when >=2 trials contribute. | If k<2, narrative synthesis used (implied; explicit threshold outside pooling: NR). |
| Direction alignment | Positive effects reflect improvement; outcomes where lower values indicate improvement are sign-reversed prior to pooling. | Examples: lower RMT indicates greater corticospinal excitability; higher MEP, FMA-UE, Barthel/MBI indicate improvement. |
| Missing SD handling | Derive missing SDs from SEs, CIs, p-values, or test statistics when possible; otherwise impute from similar studies/timepoints with documentation. | Hierarchy: derive SD from SE -> 95% CI -> p-value/t-statistic -> other reported test statistics; if not derivable, impute SD from the most similar study/timepoint/outcome definition and document the donor study/timepoint. |
| Heterogeneity | Summarize heterogeneity with I^2 and tau^2; report prediction intervals when feasible [22]. | tau^2 estimated using DerSimonian-Laird; prediction intervals were reported when k >= 3; when k < 3, prediction intervals were not emphasized due to instability. |
| Subgroup analyses | Prespecified subgroup factors: robot class; stroke stage; training dose; biomarker family; hybrid vs standalone interventions. | Subgroup meta-analysis required k >= 2 per level; otherwise results were reported narratively as prespecified. |
| Sensitivity analyses | Exclude high risk-of-bias studies; compare fixed-effect vs random-effects; exclude hybrid interventions; leave-one-out influence analyses. | Operational definition of high risk of bias: NR (based on RoB 2/ROBINS-I). |
| Meta-regression | Meta-regression will be considered when sufficient studies contribute to an outcome and covariate reporting permits. | Meta-regression was prespecified only when k >= 10 for a given outcome and covariate reporting was sufficient; otherwise not performed. |
| Multi-arm trials | Avoid double counting shared controls by combining similar eligible robot arms or splitting the shared control group evenly across conceptually distinct comparisons; document and explore in sensitivity analyses. | If multiple robot arms were conceptually similar, arms were combined using standard formulas for pooled means/SDs. If conceptually distinct, the shared control group was split evenly across comparisons (N_control divided; mean/SD unchanged) to avoid double counting. |
| Narrative synthesis | Follow SWiM guidance; group studies by biomarker family and design, then by robot class and stroke stage when possible; summarize direction using standardized effects when extractable or vote-counting by direction when pooling inappropriate [23]. | Vote-counting operational details: NR. |
| Non-randomized studies | Synthesize narratively and quantitatively only when design and outcomes support meaningful pooling; interpret cautiously due to confounding risk. | Criteria for meaningful pooling beyond ROBINS-I: NR. |

Legends: CI, confidence interval; FU1, follow-up 1 (1-3 months); FU2, follow-up 2 (>=6 months); FMA-UE, Fugl-Meyer Assessment for Upper Extremity; I^2, I-squared statistic; MD, mean difference; MEP, motor evoked potential; MBI, Modified Barthel Index; NR, not reported; RCT, randomized controlled trial; RMT, resting motor threshold; SD, standard deviation; SE, standard error; SMD, standardized mean difference (Hedges' g); SWiM, Synthesis Without Meta-analysis; tau^2, between-study variance.

**Table S2I. Missing data handling and imputation log rules**

Purpose: Provide transparent rules for deriving or imputing missing quantitative inputs and documenting decisions.

| **Data issue** | **Primary approach** | **Alternative / imputation approach** | **Documentation requirements** |
| --- | --- | --- | --- |
| Incomplete or unclear outcome reporting | Contact study authors for clarification or missing data. | NR. | Record contact attempts and resulting data clarifications (template: NR). |
| Missing SD (post or change) | Derive SD from reported SE, CI, p-value, or test statistic where possible. | If derivation not possible, impute SD from similar studies or timepoints. | Document source values, formulas used, and justification for imputation. |
| Missing change-score variance | Impute pre-post correlation when required to compute change-score SD/variance. | Explore plausible alternative correlation values in sensitivity analyses. | Report imputed correlation(s) used and results of sensitivity analyses; prespecified range not stated (NR). |
| Baseline imbalance concerns | Examine baseline imbalance descriptively. | If meaningful imbalance suspected and both can be computed, compare post-score vs change-score-based estimates in sensitivity analyses. | Document rationale for imbalance concern and which effect metric was prioritized. |
| Multiple muscles/conditions for neurophysiology | Extract trial-designated primary outcome/condition. | If not specified, prioritize a hand intrinsic target (FDI/APB) and the most commonly reported condition; choose the timepoint/condition with most complete data. | Document selection rationale and any excluded alternative conditions. |

Legends: CI, confidence interval; SD, standard deviation; SE, standard error; NR, not reported; FDI, first dorsal interosseous; APB, abductor pollicis brevis.

**Table S2J. Risk of bias, certainty of evidence, and publication bias plan**

Purpose: Document bias assessment tools, GRADE downgrade logic, and publication bias assessment thresholds.

| **Component** | **Tool / approach** | **Implementation rule (as described)** |
| --- | --- | --- |
| Risk of bias: randomized trials | RoB 2 (five domains). | Assess domains: randomization process; deviations from intended interventions; missing outcome data; outcome measurement; selection of reported result [18]. Two reviewers assessed independently with consensus resolution. |
| Risk of bias: non-randomized intervention studies | ROBINS-I. | Two reviewers(RSC and AC) assessed independently with consensus resolution [19]. |
| Certainty of evidence | GRADE, outcome-level. | Randomized evidence starts at high certainty; downgrade 1 level for serious concerns or 2 levels for very serious concerns across risk of bias, inconsistency, indirectness, imprecision, and publication bias [24]. |
| Risk of bias downgrading (GRADE) | Based on RoB 2 judgments. | Downgrade when most information comes from studies at high risk of bias or when domain-level concerns likely materially influence the estimate. |
| Inconsistency downgrading (GRADE) | Heterogeneity magnitude and CI overlap. | Consider I^2/tau^2, overlap of confidence intervals, and plausibility of a common direction of effect; use prespecified subgroup structure to interpret explainable heterogeneity. |
| Indirectness downgrading (GRADE) | PICO alignment. | Downgrade for differences in population, intervention implementation, comparator intensity, biomarker acquisition, or outcome definition relative to the review question. |
| Imprecision downgrading (GRADE) | CI width and information size. | Consider CI width and whether intervals include trivial and meaningful effects; use sample size and event information to judge optimal information size when feasible. |
| Publication bias downgrading (GRADE) | Funnel plot/small-study effects and selective reporting plausibility. | Consider funnel plot asymmetry/small-study effects and plausibility of selective reporting given outcome multiplicity and incomplete reporting. |
| Publication bias tests | Funnel plots and Egger regression. | Planned when >=10 studies contribute to an outcome [25]. |

Legends: CI, confidence interval; GRADE, Grading of Recommendations Assessment, Development and Evaluation; I^2, I-squared statistic; NR, not reported; RoB 2, Risk of Bias 2; ROBINS-I, Risk Of Bias In Non-randomized Studies - of Interventions; tau^2, between-study variance.

**Table S2K. Reproducibility checklist (computational environment and analytic transparency)**

Purpose: Itemize reproducibility elements referenced in the Methods and specify software versions used for AI-assisted checks and document generation.

| **Reproducibility element** | **Planned reporting / location** |
| --- | --- |
| Software | Analytic syntax planned to be reported for R and/or Stata in Supplementary Material 2 (Appendix S1). Primary meta-analysis software stated as R/Stata (versions: NR). AI-assisted checks and document generation used Python 3.11.2 (pandas 2.2.3, numpy 1.24.0, scipy 1.14.1, statsmodels 0.14.3, openpyxl 3.1.5); python-docx 1.2.0; LibreOffice 25.2.3.2 for rendering. |
| Statistical code | Provide full analytic syntax in Supplementary Material 2 (Appendix S1). |
| Effect size computation details | Provide demonstration of MD and Hedges' g computation and direction-flip rules. |
| Random-effects implementation | Specify tau^2 estimator, CI method, and prediction interval computation. |
| Imputation log | Document all SD derivations, SD imputations, and pre-post correlation assumptions. |

Legends: CI, confidence interval; MD, mean difference; NR, not reported; SD, standard deviation; SMD, standardized mean difference; tau^2, between-study variance.
