# Supplementary material for: Robot‐Assisted Upper‐Limb Rehabilitation After Stroke: A Systematic Review and Meta‐Analysis of Cortical Reorganization and Neuroplasticity Biomarkers
Source: Neural Plast. 2026 Jun 5;2026:9282578. doi: 10.1155/np/9282578 (PMC13238253; doi:10.1155/np/9282578)
Supplement: Supplementary file 4 — Supporting Information 4 Table S4: Sensitivity analyses for key outcomes at post‐intervention. [file NP-2026-9282578-s004.docx]

**Supplementary Material 4. Sensitivity analyses (post-intervention).**

This supplement reports sensitivity analyses for prespecified key outcomes at immediate post-intervention (post), restricted to the primary contrast (robot-assisted training vs non-robot control). Sensitivity scenarios evaluate model choice and the influence of individual studies where pooling was feasible.

Notes: Effect direction is aligned so that positive values indicate improvement; for resting motor threshold (RMT) the sign is reversed because lower thresholds indicate improvement. Random-effects models use the DerSimonian–Laird estimator with inverse-variance weighting; fixed-effect models use inverse-variance weighting. k indicates the number of effect sizes (comparisons) contributing to the meta-analysis; multi-arm trials were handled by splitting shared control groups as specified in the analysis-ready dataset.

**Table S4. Sensitivity analyses for pooled key outcomes at post (robot-assisted training vs non-robot control).**

| **Outcome** | **Scenario** | **k** | **Effect** | **95% CI** | **Note** |
| --- | --- | --- | --- | --- | --- |
| Ipsilesional RMT | Random-effects (DerSimonian–Laird) | 2 | 1.77 | [0.46, 3.08] | SMD (Hedges' g); change |
| Ipsilesional RMT | Fixed-effect | 2 | 1.84 | [1.26, 2.42] | SMD (Hedges' g); change |
| Ipsilesional RMT | Exclude high risk of bias (RoB 2 overall) |  | Not feasible |  | Not feasible: only one non-high RoB study remained (k=1). |
| Ipsilesional RMT | Leave-one-out |  | Not feasible |  | Not feasible: k<3 |
| Ipsilesional RMT | Exclude derived/imputed SD |  | Not applicable |  | All SDs were reported (no imputed SD) |
| Ipsilesional MEP amplitude | Random-effects (DerSimonian–Laird) | 2 | 0.52 | [-0.31, 1.34] | SMD (Hedges' g); post |
| Ipsilesional MEP amplitude | Fixed-effect | 2 | 0.40 | [-0.07, 0.87] | SMD (Hedges' g); post |
| Ipsilesional MEP amplitude | Exclude high risk of bias (RoB 2 overall) |  | Not feasible |  | Not feasible: only one non-high RoB study remained (k=1). |
| Ipsilesional MEP amplitude | Leave-one-out |  | Not feasible |  | Not feasible: k<3 |
| Ipsilesional MEP amplitude | Exclude derived/imputed SD |  | Not applicable |  | All SDs were reported (no imputed SD) |
| FMA-UE total | Random-effects (DerSimonian–Laird) | 3 | 4.48 | [0.33, 8.62] | MD; post |
| FMA-UE total | Fixed-effect | 3 | 3.09 | [1.13, 5.05] | MD; post |
| FMA-UE total | Exclude high risk of bias (RoB 2 overall) | 2 | 4.68 | [-1.45, 10.82] | Excluding high RoB trial (Singh, 2021); random-effects DL. |
| FMA-UE total | Leave-one-out (omit Calabrò, 2019) | 2 | 6.99 | [2.80, 11.18] | MD; random-effects DL |
| FMA-UE total | Leave-one-out (omit Singh, 2021) | 2 | 4.68 | [-1.45, 10.82] | MD; random-effects DL |
| FMA-UE total | Leave-one-out (omit Tang, 2023) | 2 | 2.27 | [0.16, 4.38] | MD; random-effects DL |
| FMA-UE total | Exclude derived/imputed SD |  | Not applicable |  | All SDs were reported (no imputed SD) |
| Barthel Index / Modified Barthel Index | Random-effects (DerSimonian–Laird) | 3 | 0.38 | [-0.81, 1.57] | SMD (Hedges' g); mixed post/change |
| Barthel Index / Modified Barthel Index | Fixed-effect | 3 | 0.12 | [-0.31, 0.54] | SMD (Hedges' g); mixed post/change |
| Barthel Index / Modified Barthel Index | Exclude high risk of bias (RoB 2 overall) | 2 | 0.33 | [-1.61, 2.26] | Excluding high RoB trial (Singh, 2021); random-effects DL. |
| Barthel Index / Modified Barthel Index | Leave-one-out (omit Singh, 2021) | 2 | 0.33 | [-1.61, 2.26] | SMD (Hedges' g); random-effects DL |
| Barthel Index / Modified Barthel Index | Leave-one-out (omit Tang, 2023) | 2 | -0.08 | [-1.23, 1.06] | SMD (Hedges' g); random-effects DL |
| Barthel Index / Modified Barthel Index | Leave-one-out (omit Wang, 2023) | 2 | 0.93 | [0.14, 1.71] | SMD (Hedges' g); random-effects DL |
| Barthel Index / Modified Barthel Index | Exclude derived/imputed SD |  | Not applicable |  | All SDs were reported (no imputed SD) |

*Legend: CI, confidence interval; DL, DerSimonian–Laird; k, number of effect sizes (comparisons); MD, mean difference; RoB, risk of bias; RoB 2, revised Cochrane risk-of-bias tool for randomized trials; SD, standard deviation; SMD, standardized mean difference (Hedges' g).*
